# Developing a bioactive glass coated dental floss: antibacterial and mechanical evaluations

**DOI:** 10.1007/s10856-023-06758-8

**Published:** 2023-10-19

**Authors:** Hazel O. Simila, Ana M. Beltrán, Aldo R. Boccaccini

**Affiliations:** 1 Institute of Biomaterials, Department of Materials Science and Engineering, University of Erlangen-Nuremberg, 91058 Erlangen, Germany; 2 Departamento de Ingeniería y Ciencia de los Materiales y del Transporte, Escuela Politécnica Superior, Universidad de Sevilla, 41011 Seville, Spain

**Keywords:** Antibacterial dental floss, Mesoporous bioactive glass nanoparticles (MBGNs), Oral health, Preventive dentistry, Tensile properties

## Abstract

In the present study, we investigated commercial dental floss coated with chitosan or chitosan + mesoporous bioactive glass nanoparticles (MBGNs) in order to determine the antimicrobial and mechanical properties of the newly fabricated flosses. Whereas these coatings showed notable ability to inhibit growth of both Gram (+) and Gram (−) bacteria after 24 h, the impact was negligible at 3 h. Furthermore, the tensile strength of the floss was improved by the addition of these layers, making it more durable and effective for cleaning between teeth. We therefore propose enhanced investigations of these composites since they demonstrate enormous potential in promoting oral health.

**Graphical Abstract:**

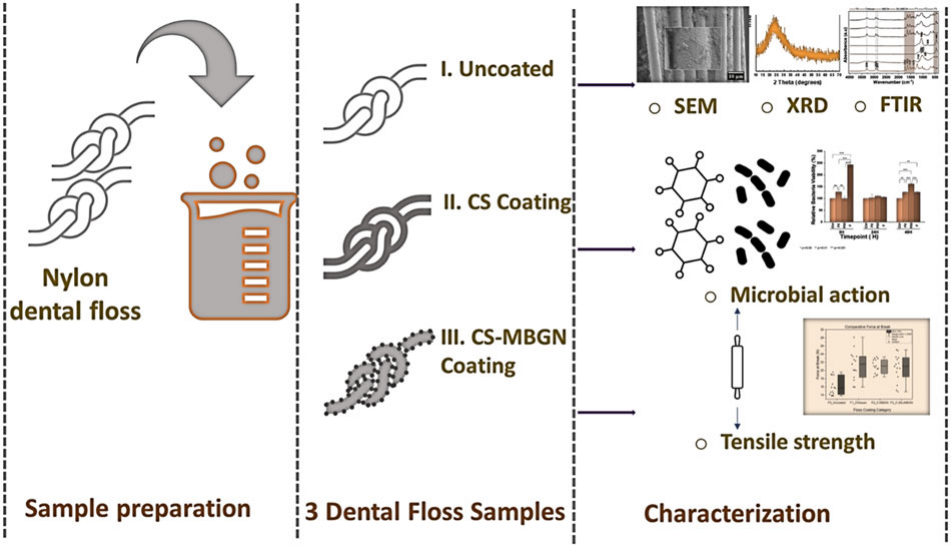

## Introduction

Flossing has been considered part of comprehensive dental hygiene since the 1800s. This practice emerged following the insufficiency of routine brushing to eliminate dental plaque from interdental surfaces, yet plaque harbors bacteria that are a contributing factor to oral diseases [1]. Unfortunately, plaque removal through flossing remains inadequate, and the magnitude of the effect is small [2]. Absence of beneficial plaque reduction from current dental floss motivates the need for further improvement, particularly by enhancing floss antibacterial properties. Emerging research is already investigating the application of coatings to standard dental floss to confer microbial action and for early caries diagnosis as well [3, 4]. At the same time, some commercial flosses are being marketed as antimicrobial. However, since most dental floss materials are based on polytetrafluoroethylene or contain wax, concerns regarding safety have been raised [5, 6]. Moreover, some of the coatings, such as those based on triclosan have been shown to lack efficacy [7], amid persistent controversy and concerns of carcinogenicity and bacterial resistance associated with triclosan [8, 9].

As such, innovation of dental floss with antibacterial compounds that are a safer choice for patients is required. Towards this aim, gold nanoparticles have been previously investigated and both the minimum inhibitory and minimum bactericidal concentrations have been determined prior to measurement of the inhibition zones associated with the experimental coated floss materials. Although the microbiology studies were comprehensive, no results were presented related to coating effectiveness or impact on floss strength and flossing efficacy [10]. Related works by Boese and Hill only target periodontal disease, whose causative agents and disease profile differ from dental caries [4, 11]. A recent patent filed by Haidar claims to develop floss for preventing both periodontal disease and dental caries, by application of coatings prepared from naturally occurring polymers and copper (Cu), silver (Ag), and lithium (Li) based nanoparticles (NPs), which possess antibacterial, antifungal, and immunostimulating effects [12]. Multiple methodologies for studying microbial inhibition were employed, and the effective prevention of the growth of different microorganisms and impairment of biofilm formation were proven. Unfortunately, these were clouded by detrimental effects on cell viability that were especially pronounced for the Cu NPs. Moreover, because the patented work only relates to silk floss materials, motivates alternative experimentation with the more commonly used nylon-based floss materials.

Besides conferment of antimicrobial capability on floss materials, supplementary roles related to desensitization by various salts, or abrasion via sodium bicarbonate [13], and prevention of gingival bleeding by coating dental floss with hemostat have been patented in the past [14]. Interestingly, although one such patent utilized chitosan derivatives as hemostatic agents [14], the investigation of the antimicrobial potential is not considered, which validates the focus of our current experiment. Ultimately, the foregoing examples shed light on the untapped potential of utilizing dental floss as a carrier for specific additives for targeted functionalities related to oral health conditions.

The specific investigation of chitosan and mesoporous bioactive glass nanoparticles (MBGNs) presents a novel strategy since independently, both chitosan and MBGNs are biocompatible and endowed with antimicrobial capabilities. Additionally, chitosan is polycationic and unique given its adhesive potential that could enhance the integrity of any ceramic-based nanoparticles that are layered on polymeric dental floss [15]. Strategies employed to coat polymeric sutures and wound dressings can be borrowed and applied to floss coating [16, 17]. Therefore, the aim of our study is to fabricate unwaxed nylon dental floss with coatings of chitosan and bioactive glass in the form of SiO_2_-CaO MBGNs. To the best of the authors’ knowledge, this approach for enhancing interproximal anticaries strategies has not been investigated before, yet it could present a promising solution to the inadequacy of current dental flossing products. Moreover, this would be a unique contribution to the emerging and vibrant area of research that investigates incorporation of sol-gel derived bioactive glasses in the field of restorative dentistry [18].

## Materials and methods

### Materials

SiO_2_-CaO (nominal composition) MBGNs were synthesized by sol-gel method according to an already published protocol [19], and characterized by bright-field transmission electron microscopy (BFTEM) with energy-dispersive X-ray analysis (EDX) (TEM, FEI Talos F200S microscope).

### Preparation of the coating

To prepare the utilized coating suspensions, 100 ml of MilliQ water (ELGA DV 25 PURELAB option R7BP) was mixed with a 1 ml of acetic acid (VWR international) and stirred briefly. 50 mg of medium molecular weight chitosan powder (75–85% deacetylation degree), (Sigma Aldrich™) was added to the 1% aqueous solution of acetic acid and stirred for an additional hour at 25 °C until clear. This yielded the primary chitosan coating solution. A 20 ml aliquot was measured from this solution to which 800 mg of MBGN particles was added and stirred gently for a further hour to yield the second coating suspension (4% w/v). A dip coater (RDC 21-K, Bungard) was used to automatically coat the unwaxed nylon floss materials (Dental Source, Kaufland, Germany) with either of the suspensions. Initially, floss samples (F) measuring 15 ± 1 cm long were cut from commercial floss roll and the tips secured with a knot to prevent unraveling of the floss fibers. The floss samples were suspended using clips attached to the lift bar of the dip coater, and lowered into 20 ml of the respective coating solution at a speed of 500 mm/min. The floss remained in suspension for 1 min before being gently withdrawn at a rate of 500 mm/min and being allowed to drip dry for one hour before transfer into petri dishes and final overnight drying inside a fume hood. Optimization of the coating procedure was based on an Edisonian approach (one-variable-at-a-time experimentation) that helped identify the ideal solvent, MBGN concentration, immersion time, and number of coatings that yielded the desired uniformity and stability of the coating as determined by scanning electron microscopy (SEM) imaging. Coated samples were separated into three groups in accordance with the type of floss sample treatment. Uncoated floss, floss coated with chitosan only and chitosan + MBGNs were coded as F0, F1, and F2, respectively. The floss samples were also weighed in an analytical balance (OHAUS CORP model PA214CM/1 scale, Nänikon, Switzerland) prior to coating. After coating application and drying, the new weight was recorded again. The difference between the final and initial weight was calculated and percentage weight change computed to reflect the amount of coating adapted onto the floss materials (*n* = 6). Further characterizations were performed by SEM-EDX (Auriga Base, Zeiss) Fourier transform infrared spectroscopy (FTIR) (IRAffinity-1S Shimadzu) and X-ray diffraction (XRD) (Miniflex 600 HR, Rigaku). The coating was subjected to further qualitative evaluation by simulating flossing on an artificial acrylic tooth model. Thus, the teeth were wetted with artificial saliva (AS) (Pharmadan, A/S Denmark), and the floss was carefully inserted into the interdental space and buccopalatal movements were performed three times at the contact area of the teeth. After these operations, the tooth surface and floss samples were investigated by SEM.

### Tensile strength testing

To determine the impact of the coatings on the floss samples, mechanical testing was performed (*n* = 15). Floss samples measuring 5 cm long were evaluated under tension using a universal tester (Instron, Germany) at a test speed of 100 mm/min.

### Antimicrobial assay

The inhibitory effect of the floss against *Staphylococcus aureus* (*S.aureus*) and *Escherichia coli* (*E.coli*) was tested by measuring the optical density (OD) of bacteria in suspension (*n* = 3). Both strains were purchased from Leibniz Institute DSMZ-German Collection of Microorganisms and Cell Cultures (Braunschweig, Germany). As a first step, the suspension of each strain was prepared by culturing in lysogeny broth (LB) medium (Roth, Karlsruhe, Germany) at 37 °C for 24 h, followed by determination of OD consistent with a value of 0.015 ± 0.002 at 600 nm, (Thermo Scientific GENESYS 30, Germany). A stock solution sufficient for immersion of all the samples was then prepared for each of the bacteria. Floss samples weighing approximately 100 ± 1 mg were sterilized by ultraviolet (UV) light irradiation for 1 h before immersion in 2 ml of the fresh bacteria stock solution. Finally, all the samples were incubated at 37 °C for 3, 24, and 48 h. At the select timepoints, a volume of 100 µl of suspension was placed in a 96 well plate and OD at 600 nm determined in a Microplate reader (7530, Cambridge Technology, Inc., Karlstad, Sweden). Each sample was measured in triplicate and the viability of bacteria was calculated by the following equation:

$${Re}lative\,viability( \% )=OD\,of\frac{Sample}{control}\times 100$$

In parallel, floss samples weighing 15 ± 1 mg were immersed in 10 ml of AS. The pH value of plain AS and that of the floss-containing AS was monitored at the commencement of the investigation, and at 3, 6, and 12 h. A digital pH meter (Jenway™ 3510, Thermo Fischer) was used for the measurements which were performed in triplicate.

## Results and discussion

### Morphological characteristics

**Fig. 1:**
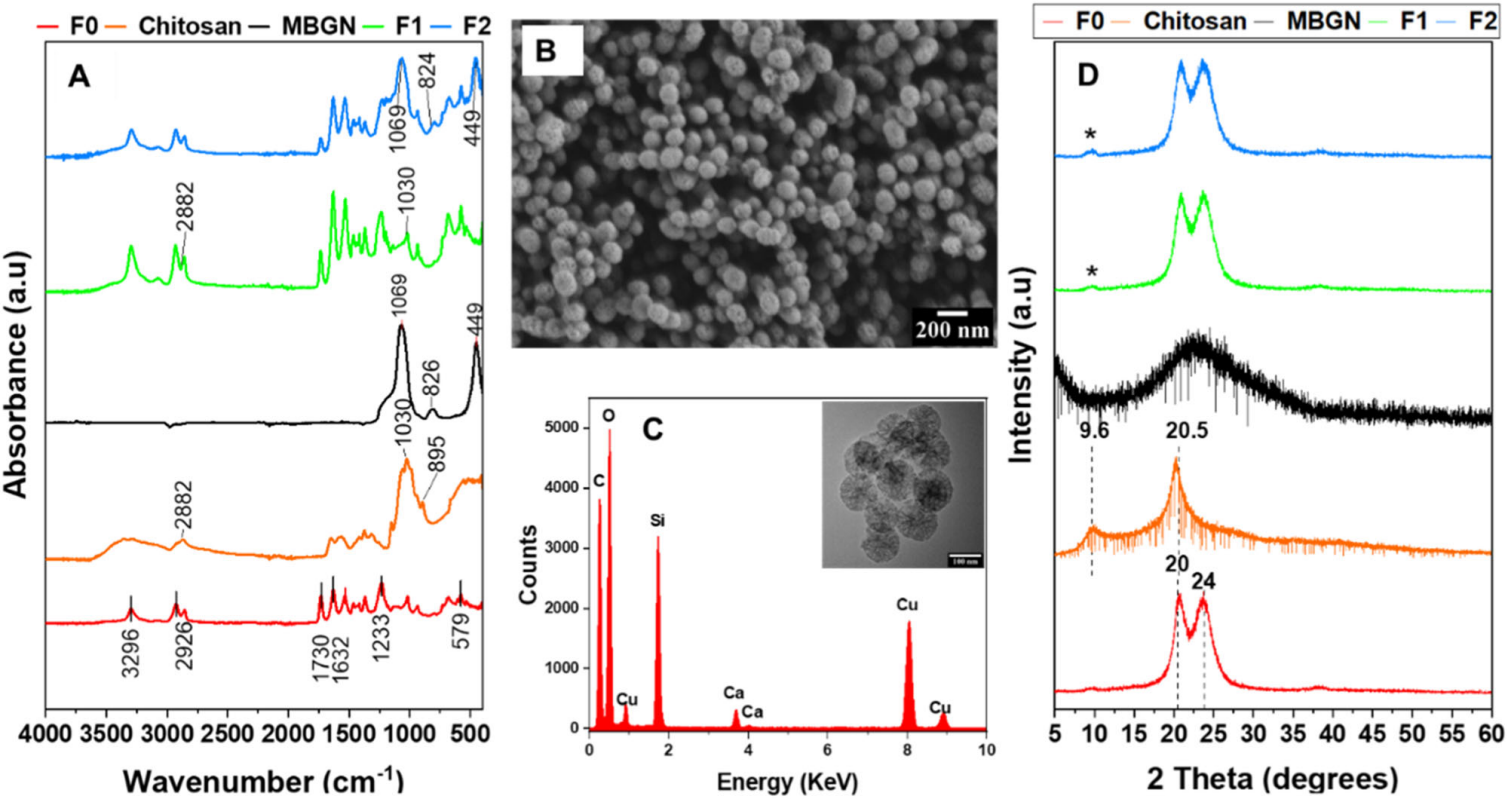
Panel representing FTIR spectra (**A**) and XRD patterns (**D**) of the precursor floss, MBGNs, chitosan, and coated flosses. SEM (**B**) and TEM-EDX (**C**) results of the MBGNs used in the coating are also shown

**Fig. 2:**
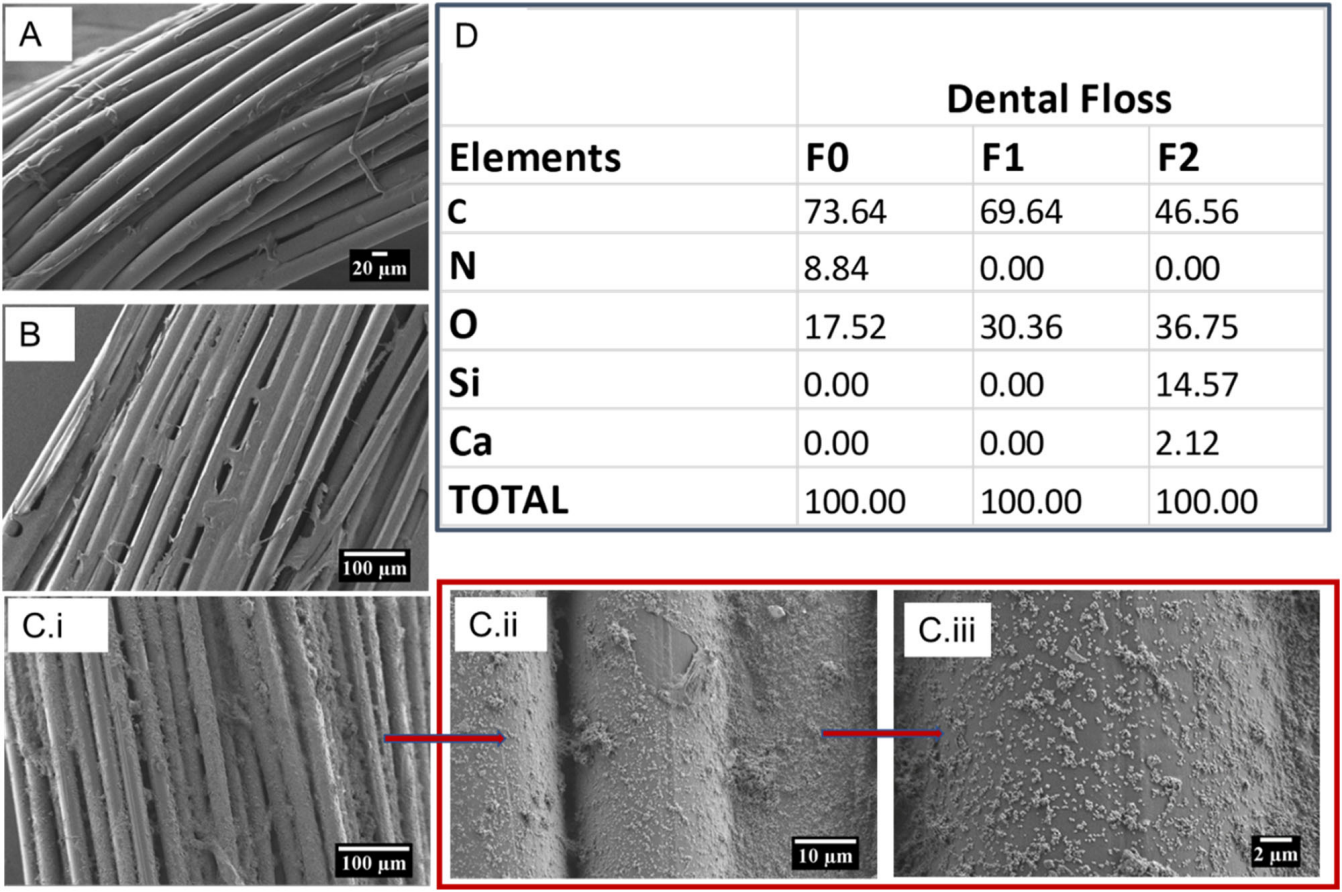
Appearance of the floss samples before and after coating as observed by SEM; F0 (**A**); F1 (**B**); and F2 at low (**C**. i), medium (**C**. ii) and high (**C**. iii) magnification. Coated and uncoated floss compositions according to EDX (**D**)

The SEM, BFTEM, and EDX data confirm the successful synthesis of SiO_2_-CaO MBGNs with the target composition of calcium and silica (Fig. 1B, C) which is in accordance with expected outcomes according to Zheng et al. [20]. Integration of chitosan and MBGNs onto floss can be adduced from the emergence of specific peaks at around 1069, 1030, 824, and 449 cm^−1^ in the FTIR spectra, which can be attributed to amine groups in chitosan, and Si-O bonding in MBGNs (Fig. 1A). Appearance of a specific peak at around 10° 2 theta angle of the XRD pattern is also characteristic of chitosan (Fig. 1D). In Fig. 2, a new layer of chitosan and also chitosan and MBGNs was detected which is consistent with results of another study that characterized metal organic complexes deposited on different threads by the Langmuir Blodgett technique [21].

**Fig. 3:**
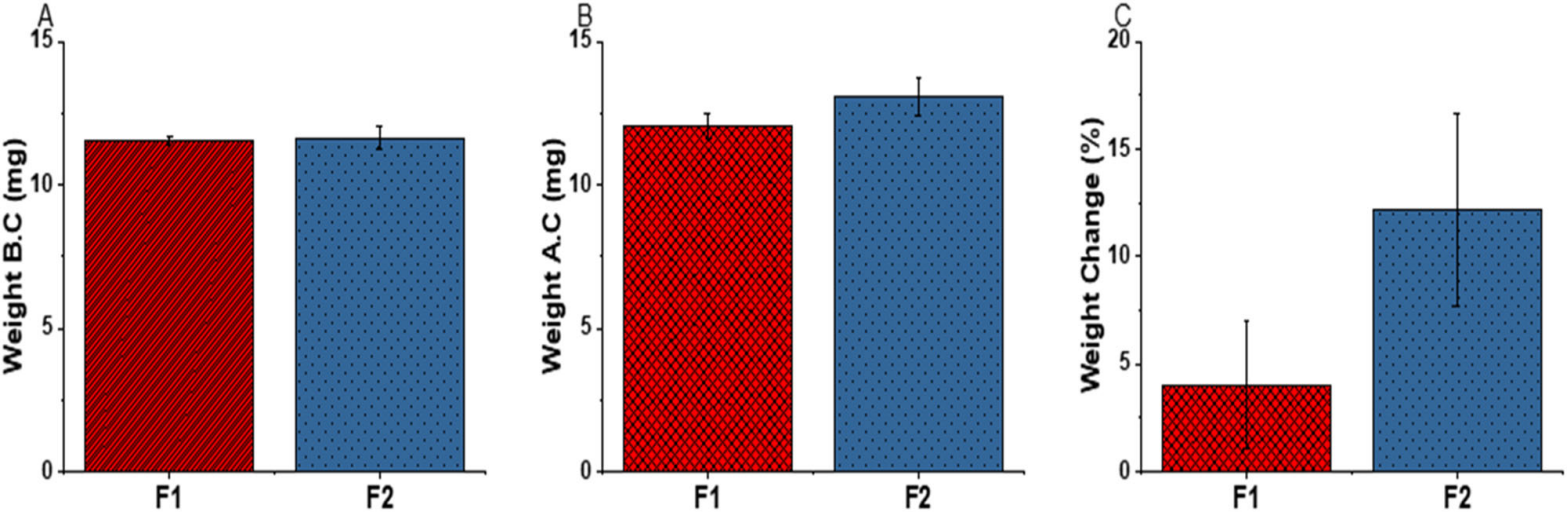
Quantification of coatings on floss materials; weight before coating (**A**); weight after coating (**B**); and percentage weight change for F1 and F2 samples (**C**). The data is reported as mean ± std dev

Further proof of the presence of the coatings was derived from the weight change after the coating procedure (Fig. 3). The results show that whereas F1 gained ~0.46 mg, which corresponds to a 24% change, the values are 1.41 mg and 73%, respectively for F2. These amounts are 2–3 times higher than the range of values for antibiotic drugs that were coated on floss materials in a different experiment targeting gum disease [11]. Besides the difference in the constituents of the coatings, the fact that the said study intentionally precoated the floss materials with poly(lactic-co-glycolic acid) to avoid entrapping drug molecules between the fibers of the braided floss, may explain the lower coating quantities obtained in the aforementioned study.

Chitosan was specifically selected in this experiment since it is a biocompatible and antibacterial agent that holds potential for enhancing the bonding between the bioactive glass nanoparticles and the nylon material [16], while acid-neutralizing and antibacterial properties of MBGNs made this mesoporous particle attractive [19].

### Mechanical properties

**Fig. 4:**
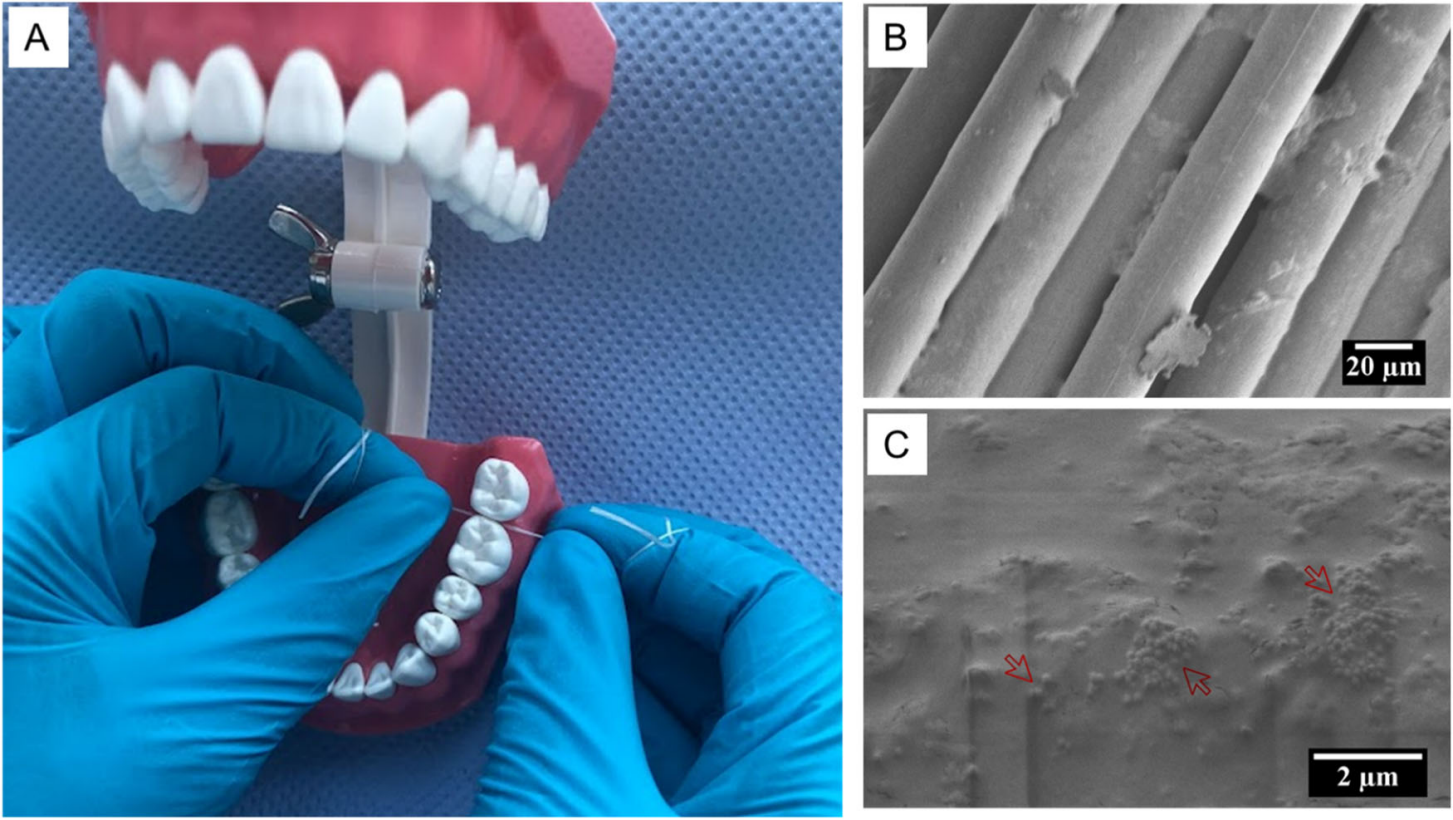
Demonstration of simulated flossing on an artificial acrylic tooth model (**A**) and subsequent appearance of the exemplary floss F2 (**B**) and tooth surface (**C**) that provides qualitative evidence of transfer of the chitosan-MBGN coating from the floss to the tooth surface. The red arrows in (**C**) denote the aggregation of sphere-shaped MBGNs

SEM observations of floss samples after simulated flossing on an acrylic tooth model (Fig. 4A) revealed similar floss fiber destruction of both the uncoated, and coated floss (Appendix 1). Additionally, the previously observed nanoparticles are distinctly less on F2 (Fig. 4B). Such loss could be interpreted to mean that the coatings were transferred into the interdental and interproximal gaps of the tooth model during flossing. Indeed, an exemplary SEM micrograph of the tooth surface reveals adaptation of spherical particles on areas of the tooth surface **(**Fig. 4C). These results bear a resemblance to findings elsewhere in which fluorescing drugs could be detected on both tooth and gingival tissues after flossing with experimental drug coated floss materials [11]. The future goal should be to utilize biological teeth and quantitatively track the transfer of the active compounds from the floss to the interdental area by multiple methods such as bright field imaging and fluorescence visualization [4].

**Fig. 5:**
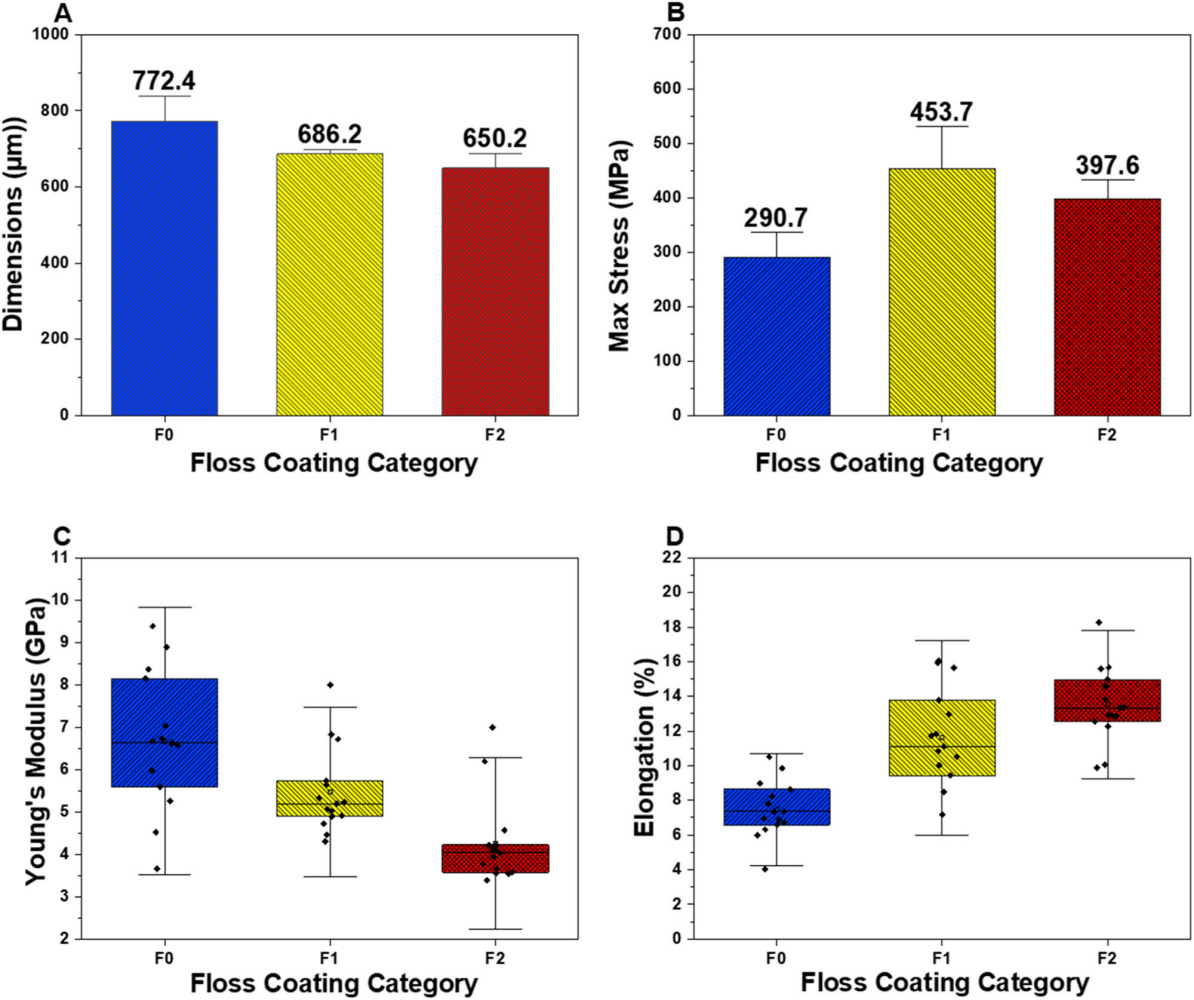
The impact of the coating procedure on floss width (**A**); tensile strength (**B**); stiffness (**C**); and displacement (**D**)

**Fig. 6:**
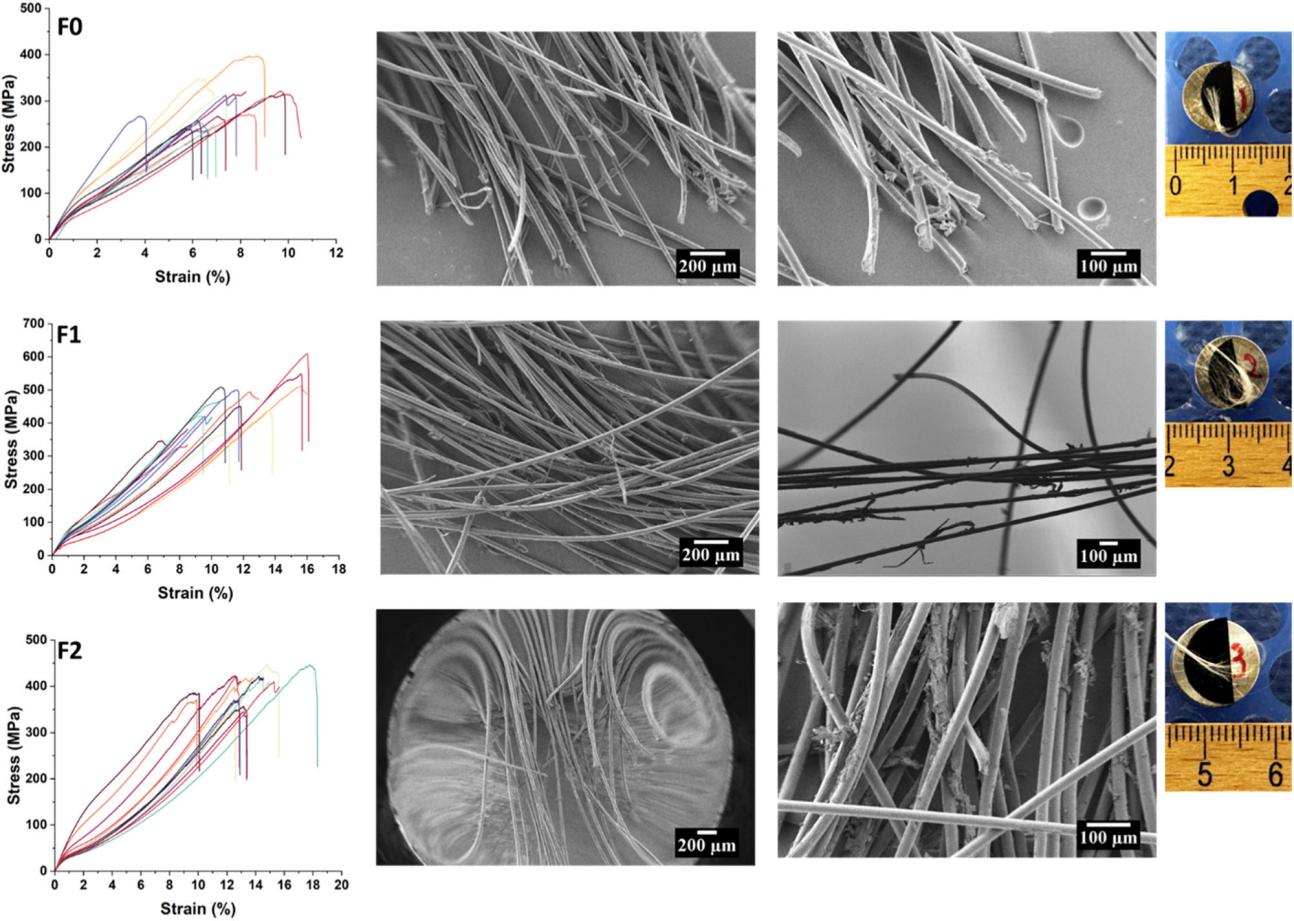
Stress–strain behavior (left), SEM (center) and photographic images (right) of the floss materials after tensile testing arranged in order from top to bottom, F0, F1, and F2

The results of the tensile testing are summarized in Figs. 5 and 6. The smaller floss diameter after coating (Fig. 5) likely arose from cation–anion interaction between the chitosan and nylon floss fibers [22]. Whereas the compacted coated floss corresponds with decreasing stiffness and increasing elongation, the strength trend is not linear and suggests a complex interplay of various factors. Naturally, the elimination of any interfilamentous spaces by compaction of the fibrils into a firm bundle equates to reduction in porous defects in the cross section of this floss, which may have strengthened F1.

The MBGNs in combination with the chitosan also provide a composite effect on the floss and this in itself should strengthen the fibers, probably by reducing any slipping motions [23] which is consistent with other studies that report strengthening of nylon fibers by ceramics [24]. Regrettably, despite their fundamental role in primary oral care, there is a dearth of information on the mechanical characteristics of dental floss [25]. Furthermore, several studies that have developed experimental floss with novel coatings fail to provide any data regarding the effects of these modifications on floss mechanical properties [4, 26].

Stress data in Fig. 5 indicate that F1 samples result in the greatest strength, although the recorded loads for both coated samples, F1 (21.78 N) and F2 (21.85 N), were almost similar. However, these values were generally lower than ranges for commercial floss that were reported by Supanitayanon et al. [25] whereby of the five floss varieties that were tested, the authors reported a minimum load of 23.7 N and maximum load of 47.4 N. In the same study, commercial floss samples resulted in displacement percentages in the range 16.44–72.43%, which are much higher than the 6–18% we currently report. General variations in test protocols and floss brands could account for the differences in values between our commercial unmodified floss and those reported in other studies. At the same time, comparison of the mean Young’s modulus values for unmodified F0 and the coated F1 and F2, which are 6.67 GPa, 5.47 GPa, and 4.26 GPa respectively, indicates a reduced tensile modulus associated with the modifications. This warrants in-depth analysis since a higher modulus is expected to correspond to greater stiffness, and stiff materials may also be expected to be stronger if other factors such as composition, flaws and grain orientation are excluded [23, 27]. From this data, it is obvious that whereas commercial floss maintains the highest rigidity, it is not the strongest.

Furthermore, among the modified floss samples, incorporating MBGNs into the chitosan appears to result in lower strength than when chitosan-only coatings are applied. This is probably where additional characteristics related to sample constitution and microstructure come into play [27]. We speculate that these differences may be due to increased interfacial defects in the presence of excess MBGNs that weaken the bond between the nylon fibers, unlike the chitosan-only F1 group in which chitosan may be strengthening this interaction. Perhaps this effect can be addressed by lowering the concentration of MBGNs in the coatings and salination of glass fillers which may enhance the interaction between nylon floss and the MBGNs and in so doing strengthen the resultant floss composite even further [28].

Exemplary images of fractured floss materials after tensile failure reveal that whereas the unmodified floss material F0 resulted in almost uniform fracture of all the fibers within the floss bundle, the threads in the F1 and F2 samples did not break and instead appear frayed and entangled in both the photographs and micrographs (Fig. 6). Such elongation of fibers interspersed with a handful of broken threads could reflect an ability to resist higher tensions by F1 and F2. This in turn suggests greater resilience, unlike the unmodified floss which also had the least elongation, suggesting release of the internal stresses with neglible elastic deformation [29] which matches the generally lower elongation computed for the unmodified floss materials and the smaller area of the stress- strain curves. Closer observation of F1 and F2 samples also reveals delamination of the chitosan membrane and agglomeration of the MBGNs between the frayed fiber bundles, respectively, in a pattern that is characteristic of loss of wax coatings from tested commercial floss materials following tensile failure [29].

### Inhibition of bacterial growth

**Fig. 7:**
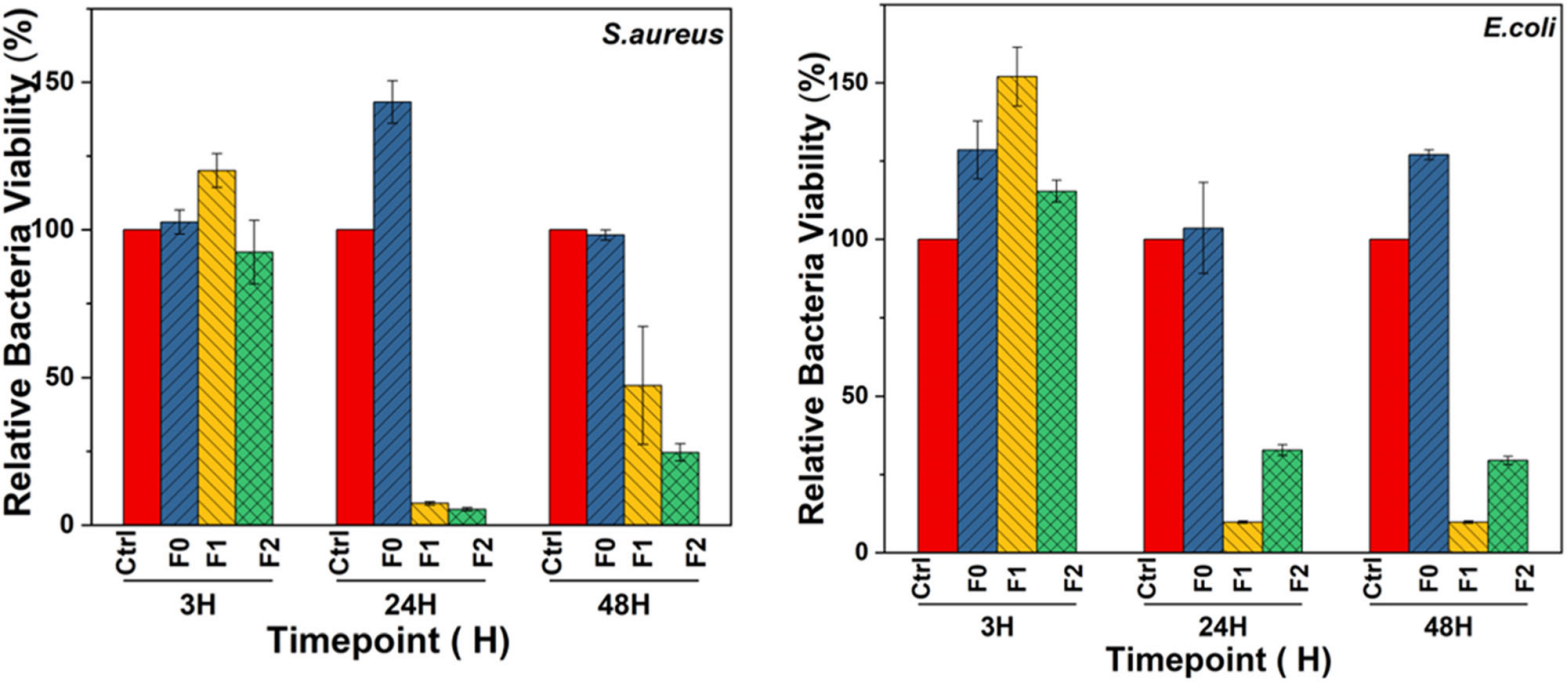
Viability of *S. aureus* and *E. coli* co-cultured with the floss materials for 3, 24, and 48 h

Independent evaluation of three different commercial floss materials validated the distinct absence of bacteria inhibition among commercial floss materials (Appendix 2). Indeed, nylon typically lacks any inhibitory effect on microbiota [30], unless the pH in the microenvironment is sufficiently acidic [31]. Follow up experiments confirm that whereas the commercial nylon floss does not have much effect on bacterial growth, chitosan-coated nylon shows a reduction of over 80% in bacterial growth (Table 1 and Fig. 7). This mirrors results of chitosan-containing-toothpaste, which was found to inhibit the growth of *S. aureus* and *E. coli* [32]. This effect has been also observed for wound dressings and was partially explained by the impact of this polysaccharide on deoxyribonucleic acid (DNA) [33]. In principle, the ability of chitosan-coated nylon to suppress bacterial growth is attributed to chitosan’s effect on the membrane-wall structure, which leads to cell damage, interference with sugar metabolism and subsequent cell death [34]. In this particular experiment, the samples coated with chitosan are likely to have inhibited the growth of *S. aureus* by disruption of the bacteria cell wall and fragmentation of the cytoplasmic membrane [34]. At the same time, chitosan causes agglomeration/flocculation of *E.coli* due to the acetylated residues present in chitosan that confer it with a cationic nature [35].

**Table 1:** Comparative bacterial viability of the coated floss materials F1 and F2, compared to the unmodified floss- F0

	Bacteria viability (%) S.D											
Time (h)	*S.aureus*						*E.coli*					
	F0		F1		F2		F0		F1		F2	
	Mean	S.D	Mean	S.D	Mean	S.D	Mean	S.D	Mean	S.D	Mean	S.D
**3**	102.6	4.1	120.3	5.7	92.4	10.7	128.6	9.2	151.6	9.3	115.3	3.5
**24**	143.3	7.2	7.4	0.5	5.4	0.5	103.3	14.5	9.8	0.4	32.8	1.7
**48**	98.2	1.5	47.3	19.9	24.7	2.9	126.9	1.6	9.8	0.4	29.5	1.3

Additionally, the MBGN coating also contributes to cell damage and suppression of bacterial proliferation by metal ion adsorption and alteration of the cell membrane’s structure [36], parallel ion release (Si, Ca) and effects on pH and osmolarity [37]. These effects seem to synergize the observed antibacterial activity of the chitosan coating and explain the enhanced antibacterial effects of the F2 group especially against *S. aureus* (Fig. 7). Indeed, our supplementary data shows slightly higher pH associated with AS exposed to the coated floss, compared to the uncoated variety (Appendix 3). The possibility to alkalize saliva is welcome since this has the promise of buffering the acidic environments associated with tooth demineralization [38]. Taken together, the potential of these floss materials to be of clinical relevance is profound. Unlike most experimental antibacterial floss materials that utilize drugs [11, 39], the utility of antibacterial ions and chitosan is highly attractive as it overcomes the antibacterial resistance associated with routine antimicrobial drug therapy.

Notably, the antibacterial effects are more prominent after at least 24 h which could be due to the need for gradual dissolution/release of the coating constituents into the bacterial cultures.

## Conclusion

We demonstrate, for the first time, the ability of chitosan-MBGNs coatings to improve the antibacterial properties of dental floss without degrading mechanical properties. Mechanical integrity of the coated floss in the presence of the newly deposited layer also enhances its utility. This study, therefore, proves that dental floss can function as a carrier of chitosan - MBGNs antibacterial compounds into the interdental space. The prospective translation of this type of device into a routine component of oral hygiene is high considering the success with which toothpaste modified with bioactive glass has been translated into a commercial product. Such products for efficacious preventive oral health care are highly encouraged compared to advanced curative interventions or rehabilitation. Undoubtedly, it is far more likely to promote compliance among users because it can be used as an over-the counter product which does not require intervention by a dental practitioner. To attain this, however, subsequent studies should address certain limitations of the current work. For example, the nylon floss could be stabilized by customized fiber holders during the coating process in order to further improve the quality of the coatings. Future studies are proposed in which specific cariogenic bacteria such as *Streptococcus mutans* and *Pseudomonas aeruginosa* should be employed. Furthermore, the device could also be tested for effectiveness against microorganisms that cause periodontal diseases. Enhanced evaluations that consider the antifungal effects as well as methods of studying inhibition of bacteria through biofilm and time kill assays are also critical. Similarly, advanced studies that quantify actual deposition of chitosan-MBGNs on the proximal surfaces, and monitor remineralization and arrest of early dental caries on natural teeth and in animal models will be more predictive of the clinical outcomes of this innovative dental cleaning device. The possibility to dope other biologically active and antibacterial ions into MBGNs (Zn, Cu, etc.) remains an interesting topic for future research. Once these aspects are addressed, and sufficient iterations undertaken to validate the concept, the logical next step will be to consider prototyping for possible testing in human subjects.

### Supplementary information

Supplementary Information
