# Supplementary material for: Developing a bioactive glass coated dental floss: antibacterial and mechanical evaluations
Source: J Mater Sci Mater Med. 2023 Oct 19;34(11):53. doi: 10.1007/s10856-023-06758-8 (PMC10587244; doi:10.1007/s10856-023-06758-8)
Supplement: Supplementary file 1 — Supplementary Information [file 10856_2023_6758_MOESM1_ESM.docx]

## **Supplementary Materials**

Appendix 1: **Similarities in appearance of the floss samples after simulated flossing**


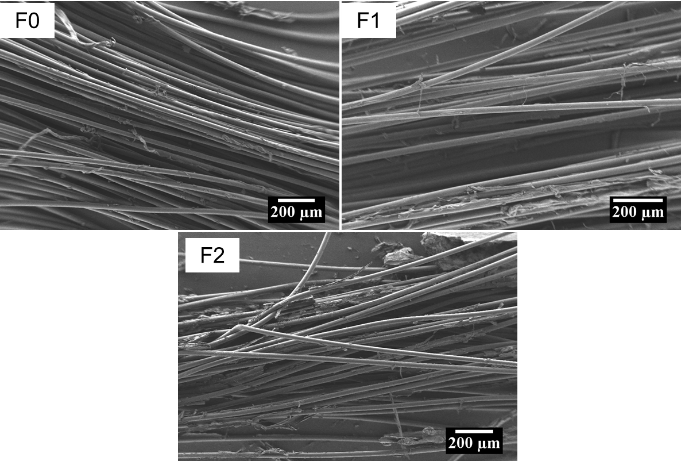


Appendix 2: Bacteria survival in the presence of 3 unmodified commercial floss materials which were either unwaxed (FO and FOO) or waxed (F).


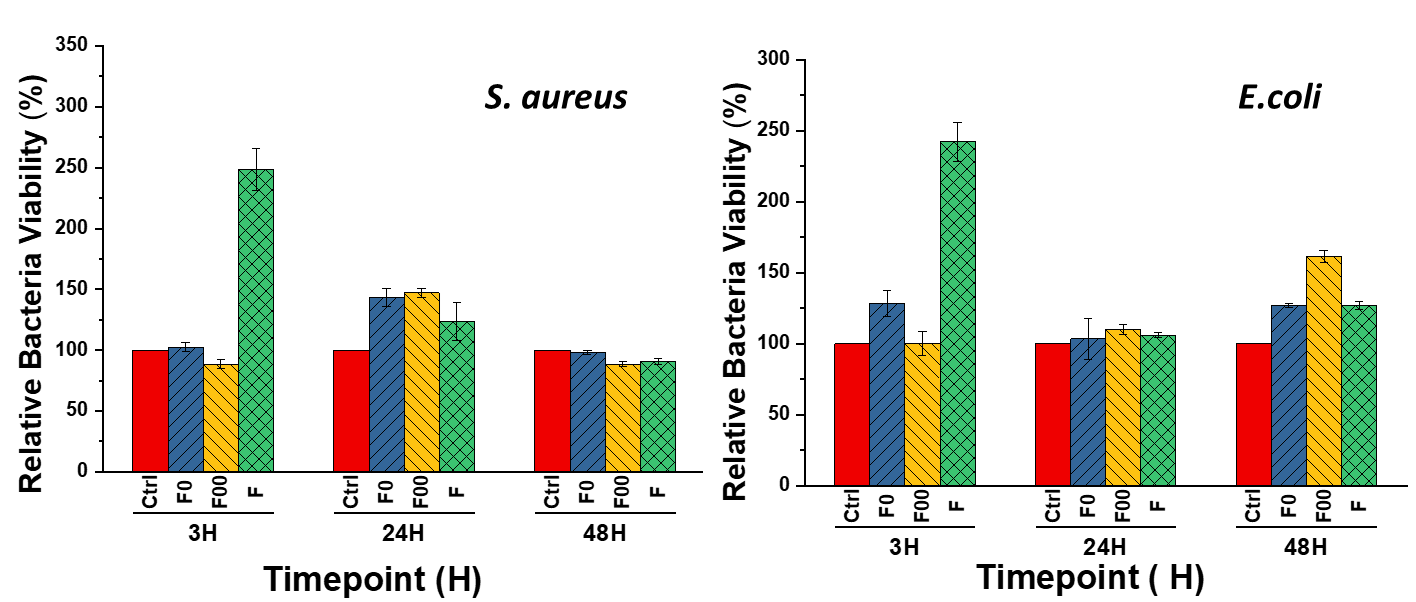


Appendix 3: Results of the effect of the various floss samples on the pH of artificial saliva following immersion for 12 hours.

*
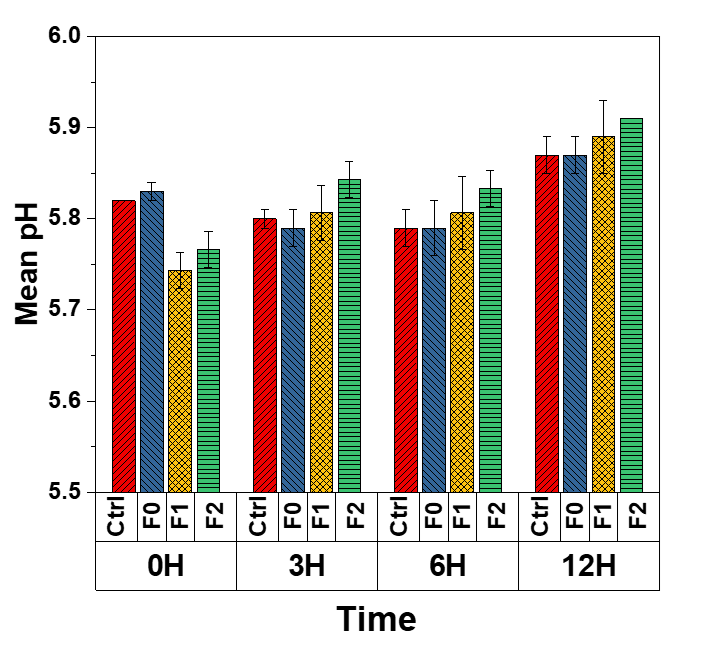
*
